# Adjuvant concurrent chemoradiation therapy (CCRT) alone versus CCRT followed by adjuvant chemotherapy: Which is better in patients with radically resected extrahepatic biliary tract cancer?: a non-randomized, single center study

**DOI:** 10.1186/1471-2407-9-345

**Published:** 2009-09-27

**Authors:** Kyu-Hyoung Lim, Do-Youn Oh, Eui Kyu Chie, Jin-Young Jang, Seock-Ah Im, Tae-You Kim, Sun-Whe Kim, Sung Whan Ha, Yung-Jue Bang

**Affiliations:** 1 Departments of Internal Medicine, Seoul National University Hospital, Seoul National University College of Medicine, Seoul, Korea; 2 Departments of Radiation Oncology, Seoul National University Hospital, Seoul National University College of Medicine, Seoul, Korea; 3 Departments of Surgery, Seoul National University Hospital, Seoul National University College of Medicine, Seoul, Korea; 4 Cancer Research Institute, Seoul National University Hospital, Seoul National University College of Medicine, Seoul, Korea

## Abstract

**Background:**

There is currently no standard adjuvant therapy for patients with curatively resected extrahepatic biliary tract cancer (EHBTC). The aim of this study was to analyze the clinical features and outcomes between patients undergoing adjuvant concurrent chemoradiation therapy (CCRT) alone and those undergoing CCRT followed by adjuvant chemotherapy after curative resection.

**Methods:**

We included 120 patients with EHBTC who underwent radical resection and then received adjuvant CCRT with or without further adjuvant chemotherapy between 2000 and 2006 at Seoul National University Hospital.

**Results:**

Out of 120 patients, 30 received CCRT alone, and 90 received CCRT followed by adjuvant chemotherapy. Baseline characteristics were comparable between the two groups. Three-year disease-free survival (DFS) rates for CCRT alone and CCRT followed by adjuvant chemotherapy were 26.6% and 45.2% (p = 0.04), respectively, and 3-year overall survival (OS) rates were 30.8% and 62.6% (p < 0.01), respectively. CCRT followed by adjuvant chemotherapy showed longer survival than did CCRT alone, especially in R1 resection (microscopically positive margins) or negative lymph node.

**Conclusion:**

Adjuvant CCRT followed by adjuvant chemotherapy prolonged DFS and OS, compared with CCRT alone in patients with curatively resected EHBTC. Adjuvant chemotherapy deserves to consider after adjuvant CCRT. In the future, a randomized prospective study will be needed, with the objective of investigating the role of adjuvant chemotherapy.

## Background

Extrahepatic biliary tract cancer (EHBTC) is a rare malignant tumor, accounting for 7500 new cases and 3300 deaths in the United States in 2005 [1]. These tumors arise from the epithelial cells of the extrahepatic bile ducts and can be divided into hilar, distal bile duct, and gallbladder origin. The type of resection and prognosis vary with anatomic location [2]. Complete surgical resection with histologically negative surgical margins has been reported to be the most important factor for the curative treatment of patients with EHBTC [3]. However, many patients are not suitable for curative surgery on presentation, and the frequency of positive resection margins has been reported to be anywhere from 9% to 74% after surgical resection [4]. In addition, loco-regional failure occurs in more than half of patients, even after complete resection [5,6]. Because of frequent local relapses and distant metastasis after curative resection, a treatment approach combining local and systemic adjuvant treatment is of interest in patients with EHBTC. The role of adjuvant treatment has not yet been established. Previous studies evaluating adjuvant radiation therapy (RT) with a variety of methods have led to conflicting results [7-10], and no large randomized trials of adjuvant concurrent chemoradiation therapy (CCRT) have been performed. However, several retrospective studies and phase II studies have reported that CCRT might have some benefits for local control, especially in the management of patients with positive microscopic margins and positive lymph nodes [10-12]. However, most of these studies have included a mixture of patients with complete and incomplete resection and have not evaluated the role of adjuvant chemotherapy after adjuvant CCRT.

Chemotherapy as a treatment modality for EHBTC has been shown to have low efficacy, and the role of adjuvant chemotherapy has not yet been thoroughly studied. A single randomized trial reported that although it did not reach the level of statistical significance, the difference in 5-year survival rates between patients who received and did not receive chemotherapy following curative resection was of large magnitude (41% versus 28%) [13]. Furthermore, in cases of advanced cholangiocarcinoma, a small randomized trial assigning 90 patients with advanced pancreatic or biliary cancer (37 with bile duct cancer) to 5-fluorouracil (5-FU)-based systemic chemotherapy or best supportive care alone suggested the benefit of chemotherapy over best supportive care alone [14]. These results suggested the possibility that 5-FU based chemotherapy could play some role in the treatment of biliary tract cancer, despite low efficacy.

Previous series have reported that distant metastasis of EHBTC occurs as frequently as local recurrence after curative resection [5,6]. Therefore, with regard to control of distant metastasis, as well as local control, adjuvant CCRT leaves something to be desired. There is still the question of whether further adjuvant chemotherapy is needed after CCRT. However, little is yet known concerning the use of CCRT alone or CCRT followed by adjuvant chemotherapy as adjuvant treatment after curative surgical resection.

The purpose of this retrospective study was to compare the clinical features, disease free survival (DFS), and overall survival (OS) between patients undergoing adjuvant CCRT alone and those undergoing CCRT followed by adjuvant chemotherapy and to evaluate prognostic factors and patterns of recurrence.

## Methods

### Patients and treatments

Of the 234 patients who had undergone radical resection at Seoul National University Hospital from January 2000 to December 2006, we collected 120 cases of invasive, non-metastatic EHBTC treated with adjuvant CCRT with or without further adjuvant chemotherapy, while 114 patients was excluded because of following causes such as no adjuvant treatment (n = 88) or adjuvant RT only (n = 12) or follow-up loss (n = 14). A total of 120 cases met the following inclusion criteria: histologically confirmed, non-metastatic adenocarcinoma of extrahepatic biliary tract except for gallbladder and periampullary cancer. This study was reviewed and approved by the Institutional Review Board of the Seoul National University Hospital. After Institutional Review Board approval, patients' medical records were reviewed for the following characteristics: age, gender, tumor markers, surgical procedures, histologic features, stage, resection margin status, radiation treatment, chemotherapy treatment, pattern of recurrence, and survival. As tumor marker, the preoperative carbohydrate antigen 19-9(CA19-9) levels were measured in most patients.

Resection procedures were classified into 4 groups: pancreaticoduodenectomy (PD) with or without pylorus preservation and segmental bile duct resection with or without hepatectomy. Lymph node dissection was done in all but 4 patients. Four patients without lymph node dissection belonged to CCRT followed by adjuvant chemotherapy group. Resection type determination was dependent on the location and extent of the tumor. All patients underwent surgical resection with the intent to cure. If a microscopic free margin could not be obtained, additional resection was attempted. But, final pathologic results reported that there were positive resection margins in 41 patients. Therefore, 41 patients had undergone R1 resection, despite additional resection. In this classification, we excluded all cases that underwent resection for palliation with gross residual tumor (R2 resection).

All pathology specimens were reviewed to determine the primary pathologic diagnosis and extent of disease. Patients were staged, based on criteria from the American Joint Committee on Cancer (AJCC), 6th edition [15].

All patients received concurrent 5-FU chemotherapy and external beam RT. 5-FU was administered at a dose of 500 mg/m^2 ^on D1, 2, 3 and on D28, 29, 30 along with 4000-5400 cGy of external beam radiation. The radiation fields were primary tumor bed and regional lymph node. The 102 patients received 40 Gy of radiation delivered as a split course of 20 Gy in 10 fractions over 2 weeks with a 2-week break between courses. The remaining 18 patients received 5040-5400 cGy of radiation without splitting course. All but 2 patients started adjuvant therapy within post-operative 3 months. Two patients who received delayed treatment due to wound problem belonged to CCRT followed by adjuvant chemotherapy group. Of 120 patients, 117 patients completed whole course of CCRT. Two patients of 3 patients who did not complete whole course were in CCRT followed by adjuvant chemotherapy group and one patient was in CCRT alone group. Also, there was no significant difference in radiation dose and method between CCRT alone and CCRT followed by adjuvant chemotherapy groups. Patients received one fraction per day, 5 days per week. The median dose per fraction was 200 cGy (range, 180-240 cGy). CCRT began a mean of 6.9 weeks after surgery (range, 4.4-14.9 weeks). The mean duration of RT was 5.8 weeks (range, 4.0-7.1 weeks). No patients died within 6 months after adjuvant CCRT.

Ninety patients received further 5-FU based adjuvant chemotherapy for 6-12 months. Adjuvant chemotherapy regimens were comprised of various combinations of 5-FU. Forty-four patients received monthly 5-FU (500 mg/m^2 ^per day for 5 days) infusion alone; 29 received 5-FU (375 mg/m^2 ^per day for 5 days) and leucovorin (20 mg/m^2 ^per day for 5 days); 16 received single oral 5-FU derivatives such as uracil-tegafur, capecitabine and TS-1; 1 received 5-FU, leucovorin, and mitomycin C. Medical oncologist gave the treatment option about additional chemotherapy after adjuvant CCRT to patient. Whether patient received additional adjuvant chemotherapy or not was dependent on patient's will. The duration of adjuvant chemotherapy depended on physician's decision.

### Statistical analysis

The primary clinical endpoints for this retrospective study were DFS and OS. Survival data were processed using the Kaplan-Meier method and were compared using the log-rank test and Cox proportional hazards regression. A *P *value of less than 0.05 was considered statistically significant. To identify differences in baseline characteristics between treatment groups, the Chi-square test and Fisher's exact test were used and continuous variables were compared using the Student *t *test.

## Results

### Patient characteristics and clinical outcomes

Patient and disease characteristics are summarized in Table 1. A total of 120 patients met the inclusion criteria. There were 89 men (74.2%). The median age was 62.4 years (range, 24.2-87.0 years). The median tumor size was 2.5 cm (range, 1.0-6.5 cm). Median numbers of dissected lymph nodes were 11.5 (range, 1-40). Positive lymph nodes were found in 35.8% of cases. Seventy-nine (65.8%) patients received R0 resection (pathologically negative margins), and 41 (34.2%) patients received R1 resection (microscopically positive margins). Twenty (16.7%) patients had well-differentiated tumors, 11 (9.2%) had at least a component of poorly differentiated tumor, and the remaining 74.1% of specimens were moderately differentiated. By the 2002 AJCC TNM staging system, 40 patients were stage I, 64 were stage II, and 16 were stage III; no patients were stage IV [15].

**Table 1:** Clinicopathologic characteristics of study population

		Total patients	CCRT	CCRT followed by adjuvant chemotherapy	*P*^+ ^value
Case		120	30	90	
Median age, years (range)		62.4 (24.2~87.0)	63.4 (24.2~74.4)	62.1 (38.8~87.0)	0.87
Men (%)		89 (74.2)	20 (66.7)	69 (76.7)	0.25
Performance	ECOG 0-1	111 (92.5)	28 (93.3)	83 (92.2)	0.73
	ECOG 2	9 (7.5)	2 (6.7)	7 (7.8)	
T stage (%)	T1	20 (16.7)	4 (13.3)	16 (17.8)	0.53
	T2	33 (27.5)	6 (20.0)	27 (30.0)	
	T3	51 (42.5)	16 (53.3)	35 (38.9)	
	T4	16 (13.3)	4 (13.3)	12 (13.3)	
N stage (%)	N0	77 (64.2)	18 (60.0)	59 (65.6)	0.58
	N1	43 (35.8)	12 (40.0)	31 (34.4)	
Stage (%)	IA	13 (10.8)	2 (6.7)	11 (12.2)	0.73
	IB	27 (22.5)	5 (16.7)	22 (24.4)	
	IIA	29 (24.2)	9 (30.0)	20 (22.2)	
	IIB	35 (29.2)	10 (33.3)	25 (27.8)	
	III	16 (13.3)	4 (13.3)	12 (13.3)	
RM status (%)	Negative	79 (65.8)	19 (63.3)	60 (66.7)	0.74
	Positive	41 (34.2)	11 (36.7)	30 (33.3)	
CA 19-9 (%)	≤ 60 U/ml	57 (47.5)	17 (56.7)	40 (44.4)	0.27
	> 60	62 (51.7)	13 (43.3)	49 (54.4)	
Tumor location (%)	Proximal	59 (49.2)	13 (43.3)	46 (51.1)	0.52
	Distal	55 (45.8)	15 (50.0)	40 (44.4)	
Histologic grade (%)	WD/MD	107 (89.2)	29 (96.7)	78 (86.7)	0.29
	PD	11 (9.2)	1 (3.3)	10 (11.1)	
Recurrence (%)	Local	22 (32.4)	5 (26.3)	17 (34.7)	0.51
	Systemic	46 (67.6)	14 (73.7)	32 (65.3)	

^+^Statistical value between CCRT and CCRT followed by adjuvant chemotherapy

ECOG, Eastern Cooperative Oncology Group; RM, resection margin; WD, well differentiated; MD, moderate differentiated; PD, poor differentiated; CCRT, concurrent chemoradiation therapy.

The median follow-up duration was 21.4 months. Death occurred in 48 of 120 patients (40.0%) during the follow-up period, all due to disease-related complications. The median DFS was 25.0 months, with a 3-year DFS of 40.8%. The median overall survival was 55.8 months, with a 3-year OS of 54.8%.

**Figure 1:**
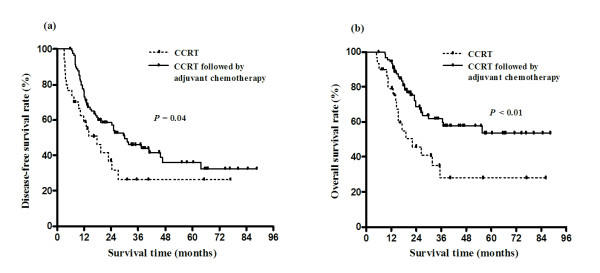
**Disease-free survival and overall survival by concurrent chemoradiation therapy (CCRT) alone and CCRT followed by adjuvant chemotherapy**. Patients treated with CCRT followed by adjuvant chemotherapy had better disease-free survival (a) and overall survival (b) compared to those treated with CCRT alone.

Out of 120 patients, 30 received CCRT alone, and 90 received CCRT followed by adjuvant chemotherapy. Median follow-up periods for the CCRT alone and CCRT followed by adjuvant chemotherapy groups were 16.6 and 23.6 months, respectively and there was no significant difference between both groups. Thirty patients who received CCRT alone showed comparatively even distribution annually. Baseline characteristics were comparable between the two groups, including age, gender, performance status, T status, nodal status, resection margin, tumor location, and other pathologic parameters (Table 1). Median numbers of dissected lymph nodes for the CCRT alone and CCRT followed by adjuvant chemotherapy groups were 12.5 (range, 1-29) and 11.0 (range, 1-40), respectively and there was no significant difference between both groups (p = 0.52). Three-year DFS rates for the CCRT alone and CCRT followed by adjuvant chemotherapy groups were 26.6% and 45.2%, respectively, with median DFS of 17.7 and 29.9 months (p = 0.04), respectively (Figure 1(a)). Three-year OS rates for the two groups were 30.8% and 62.6%, with median OS of 22.1 months and an unavailable median OS (p < 0.01), respectively (Figure 1(b)).

**Figure 2:**
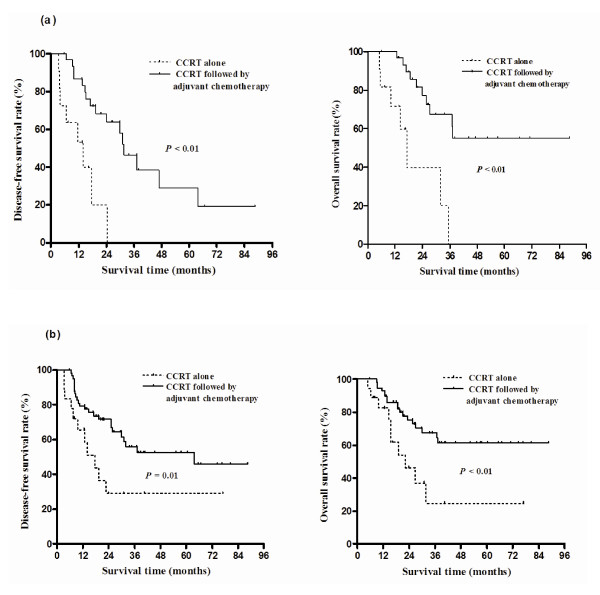
**Disease-free survival and overall survival of R1 resection (a) and negative nodal groups (b) by concurrent chemoradiation therapy (CCRT) alone and CCRT followed by adjuvant chemotherapy**. The patients treated with CCRT followed by adjuvant chemotherapy showed better clinical outcomes than those treated with CCRT alone, especially in R1 resection or negative nodal group.

For subgroups with R1 resection (Figure 2(a)) or negative nodal status (Figure 2(b)), CCRT followed by adjuvant chemotherapy led to longer DFS and OS than did CCRT alone. This same pattern was not true for patients with R0 resection or positive lymph nodes. Also, CCRT followed by adjuvant chemotherapy did not have more clinical benefit than CCRT alone in patients with T1/2 stage for DFS (p = 0.43) and OS (p = 0.50), while CCRT followed by adjuvant chemotherapy showed longer DFS (p = 0.04) and OS (p < 0.01) than CCRT alone in patients with T3/4 stage.

We analyzed subgroups according to tumor location and tumor marker CA 19-9 levels. Tumor location was classified as proximal or distal, with the cystic duct as a reference point, and CA 19-9 levels were classified as median values. For proximal tumors, CCRT followed by adjuvant chemotherapy was superior to CCRT alone with regard to DFS (p < 0.01) and OS (p < 0.01). For distal tumors, there was no difference between the groups with regard to DFS and OS. Our study also showed that CCRT followed by adjuvant chemotherapy was more effective than CCRT alone for CA 19-9 levels elevated above the median value.

### Pattern of failure

The site of relapse was evaluated for all patients. A total of 68 failures (56.6%) were observed. Loco-regional failures occurred in 22 patients (18.3%), and distant failures occurred in 46 patients (38.3%). Local relapse occurred simultaneously in 23 (50.0%) of 46 patients with distant metastasis. From a calculation of the number of metastatic sites by overlapping metastatic sites, metastatic sites included the liver in 28 patients, peritoneal cavity in 14, lung in 3, adrenal gland in 2, abdominal wall in 2, and in other areas in 2. There was no difference in the recurrence pattern between the CCRT alone group and the CCRT followed by adjuvant chemotherapy group (p = 0.51).

### Prognostic factors

On univariate analysis, significant factors for OS included elevated CA 19-9 (p = 0.048) and pattern of adjuvant treatment (p = 0.004). Histologic grade was not statistically significant, but high histologic grade tended to confer a poor prognosis (p = 0.065). CA 19-9 (p = 0.011), lymph node status (p = 0.005), pattern of adjuvant treatment (p = 0.038), and histologic grade (p = 0.029) were significant for DFS (Table 2).

**Table 2:** Univariate analysis of clinicopathologic factors associated with disease-free survival and overall survival

		No. of patients (%)	*P *value (OS)	*P *value (DFS)
Age	> 60	68 (56.7)	0.506	0.849
	≤ 60	52 (43.3)		
Sex	M	89 (74.2)	0.321	0.443
	F	31 (25.8)		
T stage	T1/T2	53 (44.2)	0.679	0.645
	T3/T4	67 (55.8)		
N stage	N0	77 (64.2)	0.223	0.005
	N1	43 (35.8)		
Stage	I	40 (33.3)	0.902	0.129
	II/III	80 (66.7)		
Resection margin	Negative	79 (65.8)	0.811	0.700
	Positive	41 (34.2)		
CA 19-9	≤ 60 U/ml	57 (47.5)	0.048	0.011
	> 60 U/mL	62 (51.7)		
Tumor location	Proximal	59 (49.2)	0.207	0.224
	Distal	55 (45.8)		
Histologic grade	WD/MD	107 (89.2)	0.065	0.029
	PD	11 (9.2)		
Angiolymphatic invasion	Yes	38 (31.7)	0.789	0.576
	No	62 (51.7)		
Perineural invasion	Yes	87 (72.5)	0.419	0.098
	No	24 (24.0)		
Adjuvant treatment	CCRT	30 (25.0)	0.004	0.038
	CCRT + AC	90 (75.0)		

OS, overall survival; DFS, disease-free survival; WD, well differentiated; MD, moderate differentiated; PD, poor differentiated; CCRT, concurrent chemoradiation therapy; AC, adjuvant chemotherapy.

On multivariate analysis, four variables for predicting survival were incorporated: CA 19-9, N stage, use of adjuvant chemotherapy and histologic grade. Of these variables, elevated CA 19-9 level, pattern of adjuvant treatment, and histologic grade were found to be significant factors for DFS (Table 3) and OS.

**Table 3:** Multivariate analysis of clinicopathologic factors associated with disease-free survival

Variable	*P *value	Hazard ratio	95% CI
CA 19-9 (≤ 60 U/mL versus 60 > U/mL)	< 0.01	0.434	0.256 - 0.735
N stage	0.17	1.430	0.862 - 2.374
Histologic grade (WD/MD versus PD)	0.01	0.345	0.162 - 0.733
CCRT alone versus CCRT followed by adjuvant chemotherapy	0.01	0.429	0.239 - 0.770

WD, well differentiated; MD, moderate differentiated; PD, poor differentiated;

CCRT, concurrent chemoradiation therapy; CI, confidence interval.

### Toxicity

Adverse effects were ranked according to the common toxicity criteria of the National Cancer Institute (NCI). In general, treatment was well tolerated. The most common acute side effects were grade 2 nausea and vomiting (43.3%). Of the 90 patients who received adjuvant chemotherapy, 16 patients (17.7%) experienced grade 2 hematologic toxicity, and 6 patients (6.7%) experienced grade 3 hematologic toxicity. Grade 1 or 2 stomatitis was recorded in 13 patients (14.4%). Grade 2 hand-foot syndrome developed in one patient.

## Discussion

Surgical resection is the only curative treatment for biliary tract cancer. Resectability is considered an important prognostic factor in many studies [16-18]. The resectability rate of EHBTC has been reported to range between 10% and 47%, despite differences in resectability rate according to tumor location [19-22]. Following complete surgical resection, the most common relapse pattern is loco-regional, with subsequent bile duct obstruction, liver failure, and recurrent sepsis [5]. Therefore, in considering postoperative local control, many studies have reported the role of RT with or without chemotherapy. However, there have been conflicting results for the role of adjuvant RT after radical resection, especially R0 resection, and no randomized controlled trial has resolved the inconsistent findings.

Many retrospective studies and small phase II studies have shown a benefit with adjuvant RT, especially for the control of microscopic residual tumor [8-10,12,23]. Oh et al. showed that adjuvant RT was useful in patients with microscopic residual tumor [23]. A recent study by Todoroki et al. revealed that an adjuvant RT group had a higher 5-year survival rate (33.9%) than a group with resection alone (13.5%), especially for patients with R1 resection [12]. Also, Todoroki et al. reported the efficient eradication of microscopic local-regional tumor residue by adding intraoperative RT to postoperative RT appeared to result in prolonging survival by preventing distant metastasis. Kim et al. reported the results of adjuvant CCRT with and without adjuvant chemotherapy after radical resection in 84 patients with EHBTC [10]. In this study, patients with microscopically positive resection margins had a 5-year survival rate of 35%, and patients with microscopically negative margins had a 5-year survival rate of 36%. This result suggested that despite no significant difference between the survival rates of the two groups, adjuvant CCRT might play a role in patients with R1 resection. Serafini et al. reported that median survival (41 months) of patients with distal cholangiocarinoma receiving adjuvant CCRT was significantly longer than that (25 months) of patients not receiving adjuvant CCRT [11].

However, some studies have shown no benefit with adjuvant RT in patients with EHBTC [2,7,24]. One prospective study showed that surgical resection and RT in 50 patients with perihilar tumors had no beneficial effect on survival or quality of survival, although the fact that lymph node evaluation was done in only 15 patients and no information was gathered concerning T stage might reduce the value of this prospective study [24]. Another small randomized trial conducted in 207 patients with pancreatic or periampullary malignancies failed to demonstrate a survival benefit for postoperative CCRT compared to surgery alone [25], but the study was limited in that there were fewer than 100 patients with periampullary cancers in this trial, only some of which were of biliary origin. Twenty percent of the patients in the treatment arm received no adjuvant treatment because of postoperative complications or refusal.

Many hospitals have carried out CCRT as a practical treatment option for patients with positive resection margins, advanced T stage, or lymph node metastasis, although this adjuvant treatment has not been demonstrated to be the standard through randomized prospective study. Our institution recommended adjuvant CCRT with or without further adjuvant chemotherapy in some patients, based on assumptions that loco-regional disease recurrence rates are high and that improving loco-regional disease control improves survival [7,12,26,27].

In this study, we reviewed 120 patients treated with adjuvant treatment after R0 and R1 resection. Adjuvant CCRT followed by adjuvant chemotherapy was significantly different with regard to DFS (p = 0.04) and OS (p < 0.01), compared with CCRT alone. Patients that had either R1 resection or negative node status experienced more benefits with CCRT followed by adjuvant chemotherapy. However, there was no significant difference in DFS and OS between the CCRT alone and CCRT followed by adjuvant chemotherapy groups for patients with R0 resection (p = 0.43, p = 0.15) or positive nodal status (p = 0.96, p = 0.23). The local failure rate was not different between the CCRT alone (5 out of 30, 16.7%) and CCRT followed by adjuvant chemotherapy (17 out of 90, 18.9%) groups. Furthermore, the rate of distant metastasis was no different between the CCRT alone (14 out of 30, 46.7%) and CCRT followed by adjuvant chemotherapy (31 out of 90, 34.4%) groups. However, even though it was not statistically significant, CCRT followed by adjuvant chemotherapy showed a tendency to prevent the development of distant metastasis.

The results of our study showed that adjuvant CCRT might be as effective in controlling microscopic residual tumor as some previous studies have suggested, and there is the possibility that further adjuvant chemotherapy could reduce recurrence, especially systemic relapse, after CCRT for patients with R1 resection. The loco-regional relapse rate in our study was lower than that seen in previous studies [5], because we classified a concomitant relapse at loco-regional and distant sites as a systemic recurrence.

Although tumor location had no prognostic impact on survival on univariate analysis in this study, patients with proximal EHBTC had better DFS and OS when treated with CCRT followed by adjuvant chemotherapy. Patients with distal EHBTC had no significant difference in survival when treated with CCRT alone or CCRT followed by adjuvant chemotherapy.

Radiosensitization with cytotoxic agents has become standard practice in the treatment of gastrointestinal malignancies. 5-FU is known to be one of the most active single agents in biliary tract cancer and is frequently used in a combined modality approach because of its potential radiosensitization effect. In general, these regimens are well tolerated. However, the number of patients treated with adjuvant RT alone in this study was small, and direct comparison was not done.

Adjuvant chemotherapy after adjuvant CCRT has not been routinely applied to patients with EHBTC and has not yet been studied. The role of further adjuvant chemotherapy remains unclear. 5-FU and gemcitabine-based regimens are used universally and are known to be active in metastatic EHBTC. 5-FU-based regimens include 5-FU alone, 5-FU in combination with other agents, uracil-tegafur, capecitabine, and S-1. In the palliative setting, these 5-FU-based chemotherapy yields response rates of 10-40% and overall survival somewhat better than best supportive therapy alone [14,28-30]. On the basis of this background, the group in our study treated with adjuvant chemotherapy showed a longer DFS and OS than did the group without adjuvant chemotherapy, especially for patients with R1 resection, patients with more than T3 or patients with negative nodal status. No beneficial effects of further adjuvant chemotherapy were shown in the lower risk patients, such as those with R0 resection or those who were less than T2. Furthermore, there was no significant difference in survival between the two groups with positive nodal status, despite the assumption that adjuvant chemotherapy would reduce local and systemic relapse and result in a survival advantage. The implications of these results should be considered. First, current nodal status simply classifies N0 and N1, regardless of the number and location of involved nodes, and cannot reflect the exact extent of disease. Second, some incomplete lymph node dissections could have been done. Third, the efficacy of 5-FU was still low, in spite of its active agency in biliary tract cancer. This suggests a need for intensification of adjuvant treatment through the use of novel agents in cases with node metastasis.

Prognostic factors such as extent of surgical procedure, T stage, nodal status, tumor location, and others have been identified in many reports [3,9,10,12,22,24,30]. In our study, the pattern of adjuvant treatment, elevated CA 19-9 level, and histologic grade were significant prognostic factors for DFS and OS on multivariate analysis.

By its nature, our study was subject to several limitations. First, the study is a retrospective series, which could have shortcomings such as selection bias. Second, adjuvant chemotherapy was not identical. We did not consider factors such as differences in efficacy between 5-FU alone and combination 5-FU therapy or duration of chemotherapy. The effect of these differences on the study outcome is unknown. Third, as a small portion of patients was included in subgroup analysis as R1 resection, our results should be interpreted with caution.

Despite these shortcomings, our study is valuable for many reasons. First, this study is one of largest reports to date comparing CCRT and CCRT followed by adjuvant chemotherapy. Second, unlike previous reports, the population in our study was characterized by a homogeneous disease representation. Previous studies looked at a combination of extrahepatic cholangiocarcinoma and other diseases, such as gallbladder and periampullary cancer. They also looked at diverse disease status, including R0, R1, and R2 resection, whereas the current study enrolled only patients with extrahepatic cholangiocarcinoma and a history of curatively radical surgery, such as R0 or R1 resection, and excluded patients with gallbladder cancer or intrahepatic cholangiocarcinoma. Therefore, in our study, CCRT with or without adjuvant chemotherapy was done only as an adjuvant treatment, not as a palliative treatment.

## Conclusion

Our work supports the superiority of CCRT followed by adjuvant chemotherapy over CCRT alone for promoting survival in curatively resectable EHBTC. Our study showed that the recurrence rate is as high as 50 percent, even if radical surgery and adjuvant treatment are employed, and this provides a strong rationale for the use of adjuvant chemotherapy after CCRT in subgroup patients, especially with R1 resection or with negative lymph node. In the future, a prospective study will be needed, with the objective of investigating the role of adjuvant chemotherapy and other active agents.

## Competing interests

The authors declare that they have no competing interests.

## Authors' contributions

KL participated in design of the study, registration and ethical approval, and review of clinical data. He also performed the statistical analysis and drafted the manuscript. DYO participated in design of the study and analyzed clinical data. EC and SH participated in patient inclusion and performed radiation therapy. JY and SW participated in patient inclusion and performed patients' operation. SAI, YTK, and YJB participated in patient inclusion and performed adjuvant or palliative chemotherapy. All authors read and approved the final manuscript.

## Pre-publication history

The pre-publication history for this paper can be accessed here:

http://www.biomedcentral.com/1471-2407/9/345/prepub
